# Reducing Procedural Waste on the Internal Medicine Wards

**DOI:** 10.1007/s11606-024-09017-w

**Published:** 2024-09-16

**Authors:** Alice Yu, Jessica Evans, Husein Moloo, Tim Ramsay, Salmaan Kanji, Mathilde Gaudreau-Simard

**Affiliations:** 1 Department of Medicine, The Ottawa Hospital, University of Ottawa, Ottawa, ON Canada; 2 Ottawa Hospital Research Institute, University of Ottawa, Ottawa, Canada; 3 Department of Surgery, The Ottawa Hospital, University of Ottawa, Ottawa, Canada; 4 Department of Pharmacy, The Ottawa Hospital, University of Ottawa, Ottawa, Canada; 5 School of Epidemiology and Public Health, University of Ottawa, Ottawa, Canada

## INTRODUCTION

Healthcare is a significant contributor to global greenhouse gas emissions, with waste responsible for 20% of this sector’s emissions.^1^ Numerous strategies aimed at reducing unnecessary waste generated from bedside procedures have been described.^2–4^ We aimed to develop a quality improvement intervention to reduce waste produced from bedside paracentesis and thoracentesis.

## METHODS

This study was performed at The Ottawa Hospital, a tertiary academic center with over 1300 beds across two campuses. This study adhered to the Standards for Quality Improvement Reporting Excellence (SQUIRE 2.0) guidelines and received an ethics waiver from our Research Ethics Board.^5^

A baseline waste audit of bedside paracentesis and thoracentesis on the internal medicine (IM) wards was performed from January to June 2023 to establish baseline waste estimates and identify opportunities for waste reduction. Interventions were developed based on findings from the first audit, in consultation with local experts and affected parties, with a goal of reducing waste by 50%. The audit was repeated from July 2023 to February 2024 after interventions were implemented. Data was collected through direct observation and by structured reporting.

### Interventions

1. Development and dissemination of a minimal supplies checklist: Residents were observed to use various functionally equivalent trays that differed in waste. An evidence-based minimal supplies checklist was created to standardize supplies and minimize waste (see Table 1).^6,7^
2. Optimization of the on-site procedure cart: The procedure cart was reorganized to include only the recommended supplies. Regular cart restocking was undertaken by unit administrators.
3. “Just in Case” to “Just in Time”: To minimize waste from over-gathering “just in case” supplies, while ensuring standard of care, additional “just in time” supplies were instead readily accessible immediately outside of the procedure areas. Unused and uncontaminated “just in time” supplies were returned to the clean utility.
4. Educational interventions: A video demonstrating implementation of the minimal supplies list was produced and distributed to internal medicine residents. Further training was communicated through weekly e-newsletters, teaching sessions, and the clinical teaching unit orientation.

**Table 1:** Minimal Supplies Checklist

Checklist item	Number required
General	
1% or 2% lidocaine, 10 mL	1
18 G blunt needle	2
22G 1–1/2″ needle	1
10 mL syringe	1
Chlorhexidine soluprep swabs	3
Sterile gloves	1
Dressing tray (includes sterile fenestrated drape and gauze)	1
Tegaderm dressing	1
A. Diagnostic paracentesis*	
Angiocath 14G	1
IV Luer lock	1
50 mL syringe	1
B. Thoracentesis, therapeutic paracentesis*	
Scalpel	1
Tape roll	1
Vacuum bottles 1 L	†
Safety needle	1

*Checklist “A” items produce less waste but can be used only for diagnostic paracentesis; “B” items can be used for both diagnostic and therapeutic paracentesis and thoracentesis

†Number varies depending on volume removed

### Outcomes and Analysis

Outcome measures included the number of wasted supplies, defined as the number of unused and discarded items gathered per procedure, as well as the per procedure waste weight. Considering that the number of fluid collection bottles used per procedure varies inherently due to patient factors, a “fixed supply waste weight metric” was calculated by subtracting the weight associated with the collection bottles from the total weight. Whitney *U* tests with continuity correction were used to compare differences between the number of wasted supplies and fixed supply waste weight for the pre- and post-intervention audits.

## RESULTS

Pre- and post-intervention audits comprised 10 procedures each (13 paracentesis and 7 thoracentesis total). Between the two audits, there was a statistically significant difference in the primary outcomes, as shown in Table 2. The proportion of total unused and wasted supplies per audit was successfully reduced by half, from 55.9% (*n* = 292) to 24.2% (*n* = 104) for the pre- and post-intervention audits, respectively.

**Table 2:** Pre- and Post-Intervention Median (IQR) Per Procedure Waste Audit Outcomes

	Pre-intervention (*n* = 10)	Post-intervention (*n* = 10)	*p*-value*
Number of wasted supplies	25.0 (19.0–43.8)	8.5 (5.0–14.0)	*p* = 0.002
Fixed supply waste weight	485.0 g (361.9–749.8)	325.3 g (238.9–351.5)	*p* = 0.012

*IQR* interquartile range, *n* number of procedures observed per audit

*Generated by Mann–Whitney *U* test

## DISCUSSION

The implementation of a minimal supplies checklist, procedure cart optimization, adoption of a “just in time” approach, and educational initiatives led to a reduction of more than 50% in wasted procedural supplies. Extrapolating from the number of thoracentesis and paracentesis that were performed by IM at our center in 2023 (*n* = 220), adoption of this initiative has the potential to reduce bedside procedure-associated waste by over 35 kg annually.

### Limitations and Strengths

A significant limitation of this work is the lack of life cycle assessments for paracentesis and thoracentesis procedures. The absence of this evidence in the literature prevented us from calculating exact carbon savings per procedure. While a larger sample size might improve the precision of our estimates, the impact and sustainability of quality improvement initiatives are best assessed through frequent cohort assessments as opposed to designing larger scale studies. The simplicity of our methods increases the feasibility of repeated assessments over time with the possibility of expansion to other procedures, units, and institutions.

### Future Directions

Further audits are planned to assess the sustainability of this initiative, with ongoing refinement of our change ideas. There is potential for this intervention to be adopted across other specialties and centers.

## Data Availability

The data is available on request to the corresponding author.
